# Entomological signatures in honey: an environmental DNA metabarcoding approach can disclose information on plant-sucking insects in agricultural and forest landscapes

**DOI:** 10.1038/s41598-018-27933-w

**Published:** 2018-07-03

**Authors:** Valerio Joe Utzeri, Giuseppina Schiavo, Anisa Ribani, Silvia Tinarelli, Francesca Bertolini, Samuele Bovo, Luca Fontanesi

**Affiliations:** 1 Department of Agricultural and Food Sciences (DISTAL), University of Bologna, Viale Fanin 46, 40127 Bologna, Italy; 2 Department of Bio and Health Informatics, Technical University of Denmark, Kemitorvet, Building 208, Room 007, 2800 Kgs. Lyngby, Denmark

## Abstract

Honeydew produced from the excretion of plant-sucking insects (order Hemiptera) is a carbohydrate-rich material that is foraged by honey bees to integrate their diets. In this study, we used DNA extracted from honey as a source of environmental DNA to disclose its entomological signature determined by honeydew producing Hemiptera that was recovered not only from honeydew honey but also from blossom honey. We designed PCR primers that amplified a fragment of mitochondrial cytochrome c oxidase subunit 1 (COI) gene of Hemiptera species using DNA isolated from unifloral, polyfloral and honeydew honeys. Ion Torrent next generation sequencing metabarcoding data analysis assigned Hemiptera species using a customized bioinformatic pipeline. The forest honeydew honeys reported the presence of high abundance of *Cinara pectinatae* DNA, confirming their silver fir forest origin. In all other honeys, most of the sequenced reads were from the planthopper *Metcalfa pruinosa* for which it was possible to evaluate the frequency of different mitotypes. Aphids of other species were identified from honeys of different geographical and botanical origins. This unique entomological signature derived by environmental DNA contained in honey opens new applications for honey authentication and to disclose and monitor the ecology of plant-sucking insects in agricultural and forest landscapes.

## Introduction

Honey is one of the few traditional food products, also used for many other purposes, that is obtained from insects. Honey is highly appreciated from its nutritive values, its sweet taste and the variety of its flavours according to its origin. It is derived by the honey bee transformation and subsequent biochemical maturation of carbohydrate containing-exudates of direct or indirect plant origin. Honey can be classified in i) blossom or floral honey, if it is mainly originated from the nectar of specialized plant structures and ii) honeydew honey, if it is mainly derived from the secretion of some trees or from the excretion of plant-sucking insects (order Hemiptera) usually feeding on the epigean parts of the plants^1^. The composition of the honey varies according to its origin. Chemical and physicochemical analyses have been used for its characterization and authentication^2–7^. In particular, differentiation between blossom honey and honeydew honey can be obtained by analyzing electric conductivity, ash content, pH, carbohydrate composition and several other parameters.

Analysis of DNA extracted from honey has been recently considered as a means useful to disclose a few characteristics of this product^8,9^. In addition, honey can be a source of environmental DNA (eDNA), that can be defined as DNA derived from the source ecological system, providing signatures of its complex biological interactions and production environment, including its origin, hive co-existing organisms (including honey bee parasites), and honey bee biological information^10^. For example, Giersch *et al*.^11^ detected the presence of *Nosema ceranae* DNA in honey suggesting that it is possible to use honey DNA as a source of information for monitoring health status of honey bee colonies. PCR amplification of targeted bee mtDNA fragments from honey-isolated DNA has been used to identify the bee species and honey bee subspecies from which the honey is produced, providing a tool for honey authentication^12,13^. The identification of baker’s yeast (*Saccharomyces cerevisiae*) from honey samples by quantitative PCR analysis of extracted DNA has been suggested as a potential assay to detect undeclared sucrose addition to honey through bee-feeding^14^. Analysis of plant DNA contained in the pollen present in the honey has been proposed as potential alternative to melissopalynology to infer its botanical origin^15,16^. Targeted sequence analyses from honey-isolated DNA have been improved using next generation sequencing (NGS) approaches. A few works have applied NGS technologies (Illumina and Ion Torrent) for metabarcoding honeys, overcoming the limits of Sanger sequencing approaches^17–20^. These studies combined the amplification of universal plant species informative regions with high massive sequencing data production to disentangle complex botanical signatures present in honeys as derived by the foraging behavior of honey bees. These works also disclosed information useful to detect the botanical (and indirectly) the geographical origin of honeys for applications on honey authentication and to infer honey bee foraging preferences opening new avenues for ecological investigation from this source of eDNA. It is therefore feasible that the honey eDNA footprint could be derived not only from plants, but also from the entomological ecosystems^21^ from which the bees obtained their carbohydrate rich nutrients. Our hypothesis is that honeydew that is transformed into honey by bees after a specific digestive process, contains useful information to reconstruct the plant-sucking insect ecosystems of the agricultural and forest landscapes of the honey bee feeding areas.

In this work, we applied a DNA barcoding approach combining Ion Torrent next generation sequencing and Hemiptera DNA markers to recover, from honeys of different origin, entomological signatures indirectly derived by honeydew produced from plant-sucking insects. The next generation sequencing approach that we used cannot be regarded as a quantitative approach even if it could approximately provide indication on relative abundance, as suggested by several studies (i.e^22–26^). This work could open new possibilities to authenticate honey and to analyse the complex entomological ecosystems of agricultural and forest landscapes using honey eDNA for retrospective and monitoring analyses.

## Methods

### Honey samples

Honey samples were provided by beekeepers or were purchased from retailers. Analysed honeys were produced in several Italian regions, in Corsica (France) and in Eastern Europe. Table 1 reports information on the investigated honeys, including their botanical or honeydew characteristics, their geographical origin and year of production.

**Table 1:** Analysed honey samples and number of total and Hemiptera reads obtained from the corresponding libraries.

Sample No.	Honey^a^	Country	Region of origin	Year of production	Number of total reads^c^	Number of Hemiptera reads
1	Oak tree honeydew honey	Italy	Lombardia (Mantova province)	2016	21605	6982
2	Honeydew honey	Italy	Trentino-Alto Adige (Bolzano province)	2016	26587	6300
3	Honeydew honey	Italy	Veneto (Verona province)	2016	22360	4392
4	Silver fir honeydew honey^b^	Italy	Emilia Romagna (Forlì province, Foreste Casentinesi)	2014	6046	2686
5	Silver fir honeydew honey	Italy	Piedmont (Cuneo province)	2017	47472	4008
6	Chestnut tree honey^b^	Italy	Piedmont (Novara province)	2015	133183	16572
7	Apple tree honey^b^	Italy	Trentino-Alto Adige (Trento, Valle dell’Adige)	2015	10054	1011
8	Linden/Lime tree (Tilia) honey^b^	Italy	Friuli-Venezia-Giulia (Udine province)	2015	6850	2098
9	Acacia honey^b^	Italy	Tuscany (Arezzo province)	2015	4802	2530
10	Eucalyptus tree honey	Italy	Calabria (Catanzaro province)	2015	28649	4900
11	Eucalyptus tree honey^b^	Italy	Sicily (Messina province)	2015	12073	2907
12	Polyfloral honey	France	Corsica	2016	38058	8933
13	Eastern Europe polyfloral honey^b^	Serbia-Romania-Croatia	Unknown	2015	129628	16572

^a^All honeys were directly provided by beekeepers except two (Eucalyptus tree honey from Calabria and Eastern Europe polyfloral honey) that were purchased from retailers.

^b^These honeys have been also analyzed by Utzeri *et al*.^20^ who reported information on the botanical origin and composition obtained by metabarcoding of a chloroplast *trnL-UAA* fragment (see also Table S2 for detailed information for all honeys).

^c^The difference between the total number of reads and the number of Hemiptera reads determines the number of *A*. *mellifera* reads (almost 99% of the remaining reads).

### Hemiptera PCR primer design

All Hemiptera mitochondrial cytochrome c oxidase subunit 1 (COX1 or COI) gene entries were retrieved from GenBank/EMBL (May 2017). Gene sequence alignment was obtained using MUltiple Sequence Comparison by Log- Expectation (MUSCLE) tool (http://www.ebi.ac.uk/Tools/msa/muscle/) and PCR primers (forward: 5′-TGGAWCAGGAACAGGATGAAC-3′; reverse: 5′-AAATGAARTTGATTGCTCCTA-3′) were selected in the most conserved regions to amplify a fragment of 135–140 bp across the main plant-sucking Hemiptera families. Supplementary Fig. S1 reports the alignment of the corresponding sequence region of a few representative plant-sucking Hemiptera species of different suborders and families. Supplementary Fig. S2 reports the alignment of the *Cinara cedri* COI sequence with the corresponding region of *Apis mellifera*. Supplementary Dataset S1 reports the alignments of the forward and reverse primers with Hemiptera sequence entries retrieved from EMBL/GenBank (June 2017) obtained with BLASTN (limited to 5000 entries).

### DNA extraction from insects

To test PCR primers and optimize cycling conditions for use of honey DNA, DNA was extracted from a few insects. Two honey bees (*A*. *mellifera ligustica*; already dead) were collected from a hive in Bologna province (Italy). Other samples of Hemiptera species of different suborders [*Issus muscaeformis*, *Metcalfa pruinosa* (Auchenorrhyncha); *Halyomorpha halys* (Pentatomidae); *Aphis craccivora*, *Cinara cedri*, *Cinara cupressi*, *Cinara pectinatae*, *Myzus persicae* (Sternorrhyncha)] were collected in Emilia Romagna region (Italy). DNA extraction was carried out on whole insect specimen using the Wizard ® Genomic DNA Purification Kit (Promega) following the manufacturer’s instructions for animal tissues.

### DNA extraction from honey samples

DNA extraction from honeys was carried out following the procedures described in Cheng *et al*.^8^ and Soares *et al*.^27^ with several modifications. Briefly, aliquots of 12.5 g of honey were placed in 4 different Falcon tubes, for a total of 50 g of starting material. Then, 40 mL of ultrapure water was added to each tube and then vortexed for 10 s followed by an incubation at 40 °C for 1 min. This step was repeated for 10 times. Then the Falcon tubes were centrifuged for 25 min at 5000 g at room temperature. The supernatant was then discarded. The pellet was resuspended in 5 mL of ultrapure water and the content of the four Falcon was merged and further diluted with ultrapure water to reach 45 mL. The combined Falcon tube was centrifuged for 25 min at 5000 g at room temperature and the supernatant was discarded. The pellet was resuspended in 0.5 ml of ultrapure water and transferred in 1.5 ml tube that was stored at −20 °C till the subsequent DNA extraction steps.

Next, one mL of CTAB extraction buffer [2% (w/v) cetyltrimethylammoniumbromide; 1.4 M NaCl; 100 mM Tris-HCl; 20 mM EDTA, pH 8; 5 μL of RNase A solution (10 mg/mL) pre-incubated for 10 min at 60 °C] and 30 μL of proteinase K (20 mg/mL) were added to the pelleted honey materials. Subsequent incubation was at 65 °C for 90 min with gentle mixing. Samples were cooled to room temperature and then centrifuged for 10 min at 16,000 g. A total of 700 μL of supernatant was transferred in a tube containing 500 μL of chloroform/isoamyl alcohol (24:1) and vortexed for 30 s. This step was followed by a centrifugation for 15 min at 16,000 g at room temperature. The supernatant was transferred in a new 1.5 mL tube and the DNA was precipitated in two steps: the first with 500 μL of isopropanol and the second with 500 μL of ethanol/water 70:30 (v/v). The pellets were rehydrated with 30 μL of sterile water and stored at −20 °C till PCR analyses.

### PCR and qPCR using Hemiptera primers

PCR amplifications were carried out using DNA extracted from the listed insect species and from DNA extracted from the collected honey samples. PCR was performed on a 2720 Thermal Cycler (Life Technologies) in a total volume of 20 μL with 2× of the Kapa HiFi HotStart ReadyMix PCR kit (Kapa Biosystems, Boston, Massachusetts, USA), 50 ng of template DNA and 10 pmol of each primer with the following PCR profile: initial denaturation step at 95 °C for 5 min, then 35 cycles of 30 s at 95 °C, 30 s at different annealing temperatures as described below, 30 s at 72 °C), followed by a final extension step at 72 °C for 5 min. Amplified DNA fragments were electrophoresed in 2.5% agarose gels in TBE 1× buffer and stained with 1× GelRed Nucleic Acid Gel Stain (Biotium Inc., Hayward, CA, USA).

Considering the need to amplify DNA from Hemiptera species isolated from honey that also contains DNA of *A*. *mellifera*^9,12,13^, PCR analyses were carried out using different annealing temperatures (from 54 to 63 °C) in order to determine the temperature at which Hemiptera DNA preferentially amplifies over *A*. *mellifera* DNA. To that end, amplification reactions were tested with DNA of the sampled Hemiptera species and *A*. *mellifera*. This empirical evaluation identified 58 °C as the temperature that could potentially maximize amplification from Hemiptera species with a reduced efficiency against honey bee DNA. To confirm that the designed primers amplified the expected COI sequence, amplicons obtained from DNA extracted from the indicated insect species were sequenced with the BrightDye® Terminator Cycle Sequencing Kit (NIMAGEN, Nijmegen, the Netherlands) and a capillary sequencer (ABI3100 Avant, Life Technologies). Amplicons obtained from the honey DNA were used for Ion Torrent sequencing (see below).

qPCR was carried out to further evaluate primer efficiency on the DNA obtained from different hemipters (from which DNA was extracted from individual subjects; see above), *A*. *mellifera* and honey DNA (Supplementary Table S1). For each reaction, 2× Kapa SYBR Green PCR reagent, 10 µM forward primer, and 10 µM reverse primer were used in a total volume of 19 μL. To each reaction, 1 μL of template DNA was added to make a final volume of 20 μL. The qPCR was performed on an ABI 2000 analyzer (Life Technologies) with the following program: 95 °C for 10 min; then 95 °C for 15 s and 58 °C for 1 min repeated for 40 cycles. Absolute changes in amplification (Ct) were measured on DNA template at the same concentration (6 ng/µL) and using 1:10 and 1:100 dilutions for each sample. Amplification efficiency of the different DNA samples is reported in Supplementary Table S1. Each analysis was done in triplicate. Primers showed a higher efficiency on *M*. *pruinosa* than on *A*. *mellifera*, as expected from the empirical analysis reported above. Amplification efficiency was higher on *M*. *pruinosa* than in the other tested hemipters. Efficiency was low on honey DNA suggesting that in complex DNA matrices it is difficult to translate efficiency reported on individual DNA samples.

### PCR for plant metabarcoding analysis

Honey-extracted DNA was analysed to obtain information on the botanical composition of the investigated samples that is mainly derived by their pollen content. Plant metabarcoding analysis was carried out by amplifying a chloroplast *trnL-UAA* fragment using primers reported by Taberlet *et al*.^28^ (forward: 5′-GGGCAATCCTGAGCCAA-3′; reverse: 5′-CCATTGAGTCTCTGCACCTATC-3′) as already described by Utzeri *et al*.^20^. Amplicons obtained from the honey DNA were used for Ion Torrent sequencing (see below).

### Ion torrent sequencing

Ion Torrent sequencing was carried out following Bertolini *et al*.^23^ with a few modifications. Briefly, PCR products obtained from each honey DNA sample using Hemiptera-specific primers and chloroplast *trnL-UAA* primers and were purified with ExoSAP-IT^®^ (USB Corporation, Cleveland, Ohio, USA) and then sequenced using the Ion Torrent PGM (Thermo Fisher Scientific Inc.). A total of 26 libraries (13 for the Hemiptera-specific primer derived amplicons and 13 for the *trnL-UAA* amplicons) were produced by end-repair and ligation of the DNA fragments with a specific barcode using the Ion Xpress^TM^ Plus Fragment Library and Ion Xpress™ Barcode Adapters 1–32 kits (Thermo Fisher Scientific Inc.). Each library was quantified with the Ion Library Quantitation kit (Thermo Fisher Scientific Inc.) by qPCR with the StepOnePlus™ Real-Time PCR System (Thermo Fisher Scientific Inc.). Seven and six barcoded libraries derived from Hemiptera-specific primer amplicons and from *trnL-UAA* amplicons were pooled and sequenced in three different Ion 318 v2 chips and one Ion 316 v2 chip (Thermo Fisher Scientific Inc.; two Ion 318 chips for the two Hemiptera pooled groups of libraries, one Ion 318 chip and one Ion 316 chip for the *trnL-UAA* pooled groups of libraries). Libraries were first clonally amplified by emulsion PCR with the Ion PGM^TM^ Hi-Q^TM^ OT2 kit and sequenced following the manufacturer’s instructions using the Ion PGM^TM^ Hi-Q^TM^ Sequencing kit.

### Next generation sequencing data analyses

Reads obtained from Ion Torrent sequencing of the produced libraries were first processed with the Torrent Suite v.4.6 on the Ion Torrent Server (Thermo Fisher Scientific Inc.). Then, obtained FASTQ files were quality checked using *fastqc* tool (http://www.bioinformatics.bbsrc.ac.uk/projects/fastqc/). Briefly, reads were separated according to their barcode and polyclonal and low-quality sequences were eliminated. Adapters and low quality 3′-ends were trimmed from the filtered reads. After this automated processing, reads were trimmed at their 5′ and 3′ end using the *trim* function of HOMER (http://homer.ucsd.edu/homer/ngs/). Retained read sequences were at least 50 bp long with a quality score equal or higher than Q20. The BLAST + suite (version 2.6.0, January 09, 2017; ftp://ftp.ncbi.nlm.nih.gov/blast/executables/blast+/LATEST/) was used for sequence identification. Trimmed Hemiptera-primer specific amplicon derived-reads were analysed with BLASTN against a customized Hemiptera COI sequence database composed of 89,580 different COI sequence entries downloaded from GenBank (https://www.ncbi.nlm.nih.gov/genbank/; May 2017). Reads matching a reference sequence with at least 97% identity and 95% coverage to 100% were assigned the associated taxonomic identification. Identification of different within-species mitotypes was obtained by aligning reads matching the same COI species-specific sequences (and derived from the same species). Alignment was obtained with *bwa* v.0.7.11^29^ according to the *aln* algorithm^30^ (http://bio-bwa.sourceforge.net/) using default parameters and customized scripts were used to automatically retrieve different mitotype sequence information. To overcome potential biases derived by sequencing errors, mitotypes were considered reliable if identified in at least 20 different reads and were already reported in other GenBank entries or that accounted at least 5% of reads assigned to the same species.

Trimmed *trnL-UAA* reads were analysed with BLASTN against a customized database constructed from a total of 78,496 different plant species. Details of the database and data analyses have been already described in Utzeri *et al*.^20^.

Proportion of reads assigned to different Hemiptera or plant species were not regarded as quantitative measures. Following Keller *et al*.^26^, abundance of reads was interpreted as categorical (e.g. high abundance, medium abundance and low abundance) using the relative proportion (frequency) of reads over all sequenced reads without any linear association. We considered high abundance Hemiptera species or botanical groups if their overall filtered reads of that library were >30%, medium abundance when their filtered reads were from 5 to 30% and low abundance when their filtered reads were <5%.

### Data availability

The datasets generated and analysed during the current study are available in the EMBL-EBI European Nucleotide Archive (ENA) repository http://www.ebi.ac.uk/ena, with the project accession number PRJEB25706.

## Results

### Botanical signature of the analysed honeys

To confirm the nectar origin of the blossom honeys and obtain other information on the botanical composition of the areas in which the honey bees collected nectar and honeydew during their foraging activities (that could then useful for a comparative analysis with the hemipter signature; see below), we evaluated the botanical signature of the investigated honeys using a metabarcoding approach^20^. Information on the plant taxa that we identified from the analysis of the chloroplast *trnL-UAA* fragment reads is included in Supplementary Table S2. Parts of these data have been already reported in Utzeri *et al*.^20^.

Almost all honeydew honeys showed the highest proportion of reads that were from *Castanea* spp. (i.e. chestnut tree), which is the most pollinic species that is widely present in the areas where these honeys were produced. Silver fir and oak are anemophilous species that do not produce nectar and for this reason they are not visited by honey bees. This might be the main reason that silver fir forest honeydew and oak honeydew honeys reported a small proportion of reads assigned to *Abies* spp. and *Quercus* spp., respectively. The honeydew honey produced in Veneto region showed the highest number of reads from *Trifolium* spp. What we obtained from the analysed honeydew honeys seems to reflect, at least in part, the inconstancy and heterogeneity in terms of pollen content that honeydew honeys usually show^3,31^ that, in turn, may affect their botanical signature.

Five unifloral blossom honeys (the chestnut tree, apple tree, acacia and the two eucalyptus tree blossom honeys) reported a plant signature that was consistent with their prevalent botanical origin (Supplementary Table S2). That means that for these unifloral blossom honeys the plant species from which these honeys took their name showed a high abundance of assigned reads to these species. The chestnut tree blossom honey showed the highest number of reads (65.8%) that matched *Castanea* spp. reference sequences. The apple tree blossom honey showed the highest number of reads (34.8%) matching the botanical group of the Family Rosaceae that also contains *Malus* species, followed by reads assigned to the genera *Prunus* (26.6%) and to the family Salicaceae (13.5%). Acacia honey showed the prevalence of *Robinia pseudoacacia* reads (41.3%), followed by reads matching *Castanea* spp. (12.4%) and several plant groups of the family Rosaceae. The two eucalyptus tree blossom honeys showed the largest number of reads matching a plant reference sequence of the subfamily Myrtoideae, that contains eucalyptus species (about 56% and 47% for the Calabrian and Sicilian honeys, respectively), followed by reads matching *Asparagus acutifolius* and *Castanea* spp. and several other plants common in the Mediterranean regions. The linden tree honey matched Fagaceae reference sequences (96.1%). *Tilia*, as botanical group, accounted 0.2% of reads. *Tilia* pollen is known to be under-represented in honey and this is confirmed by our results. The French polyfloral honey produced in Corsica reported the most frequent reads (approximately 50%) assigned to *Castanea* spp. followed by other plants that are common in the Mediterranean maquis. The botanical signature of the Eastern Europe polyfloral honey was quite heterogenous with a large frequency of *Prunus* reads (43.5%) followed by Rosaceae (11.7%), Salicaceae (10.3%), *Acer* (8.1%), *Glycine max* (8.0%) and many other species.

### Hemiptera signature in the analysed honeys

**Figure 1:**
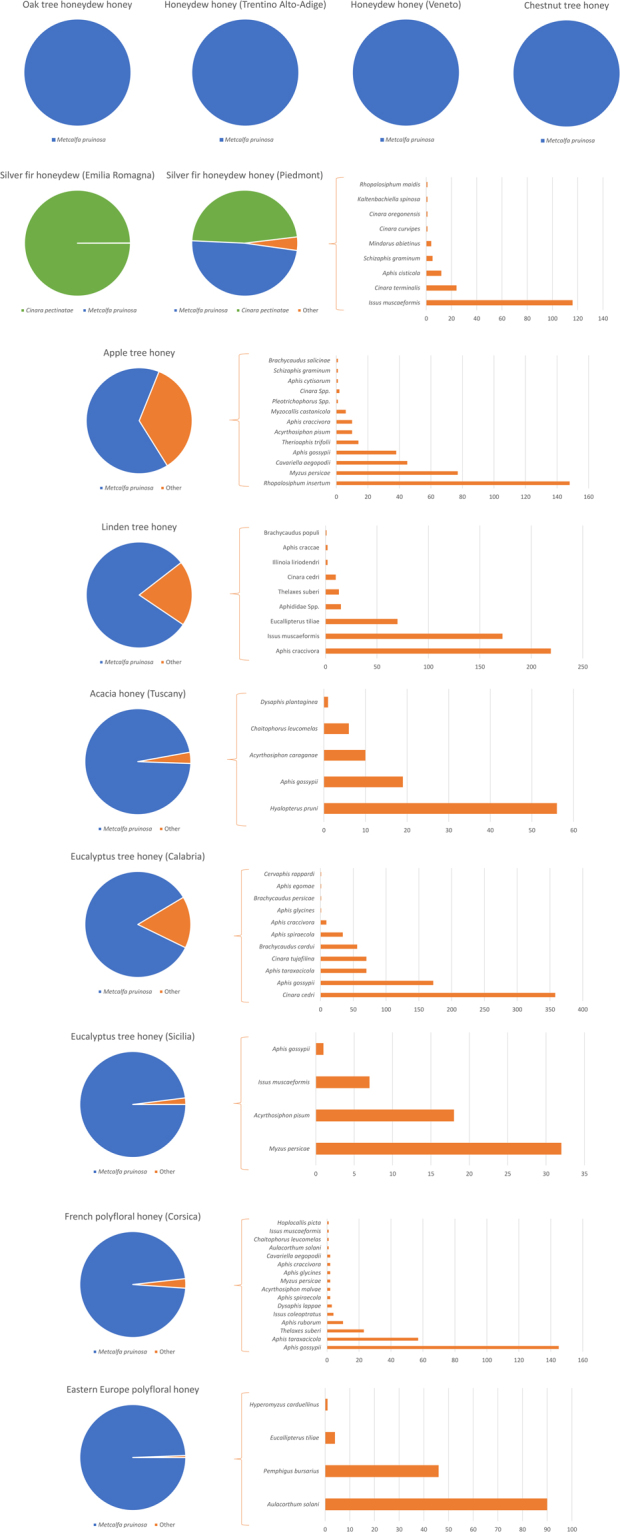
Proportion of reads from different Hemiptera species in the analysed honey samples.

Our Hemiptera-specific primers produced an amplified band corresponding to the expected size of about 140 bp in all honey samples (data not shown). Subsequent Ion Torrent PGM sequencing produced a total of 148,618 filtered reads attributed to Hemiptera species (30.5% of total reads). Its distribution in the different honey-derived libraries is reported in Table 1. The proportion of Hemiptera reads ranged from about 10% (apple tree honey) to 53% (acacia honey) confirming efficient amplification of Hemiptera DNA. All analysed honeys were positive for the presence of Hemiptera DNA, even when they were not labelled as honeydew honeys (Fig. 1 and Table 2).

**Table 2:** Hemiptera species identified in the investigated honeydew and blossom honeys.

Type of honey	Honey	Hemiptera species	No. of reads	Abundance category^a^
Honeydew honeys	Oak tree honeydew honey	*Metcalfa pruinosa*	6982	High
	Honeydew honey (Trentino Alto-Adige)	*Metcalfa pruinosa*	6300	High
	Honeydew honey (Veneto)	*Metcalfa pruinosa*	4392	High
	Silver fir honeydew honey (Emilia Romagna)	*Cinara pectinatae*	2097	High
		*Metcalfa pruinosa*	1	Low
	Silver fir honeydew honey (Piedmont)	*Metcalfa pruinosa*	1944	High
		*Cinara pectinatae*	1899	High
		*Issus muscaeformis*	116	Low
		*Cinara terminalis*	24	Low
		*Aphis cisticola*	12	Low
		*Schizaphis graminum*	5	Low
		*Mindarus abietinus*	4	Low
		*Cinara curvipes*	1	Low
		*Cinara oregonensis*	1	Low
		*Kaltenbachiella spinosa*	1	Low
		*Rhopalosiphum maidis*	1	Low
Blossom honeys	Chestnut tree honey	*Metcalfa pruinosa*	16572	High
	Apple tree honey	*Metcalfa pruinosa*	657	High
		*Rhopalosiphum insertum*	148	Medium
		*Myzus persicae*	77	Medium
		*Cavariella aegopodii*	45	Low
		*Aphis gossypii*	38	Low
		*Therioaphis trifolii*	14	Low
		*Acyrthosiphon pisum*	10	Low
		*Aphis craccivora*	10	Low
		*Myzocallis castanicola*	6	Low
		*Pleotrichophorus sp*.	1	Low
		*Cinara sp*.	2	Low
		*Aphis cytisorum*	1	Low
		*Schizaphis graminum*	1	Low
		*Brachycaudus salicinae*	1	Low
	Linden tree honey	*Metcalfa pruinosa*	2026	High
		*Aphis craccivora*	219	Medium
		*Issus muscaeformis*	172	Medium
		*Eucallipterus tiliae*	70	Low
		*Aphididae spp*.	15	Low
		*Thelaxes suberi*	13	Low
		*Cinara cedri*	10	Low
		*Illinoia liriodendri*	2	Low
		*Aphis craccae*	2	Low
		*Brachycaudus populi*	1	Low
	Acacia honey	*Metcalfa pruinosa*	2594	High
		*Hyalopterus pruni*	56	Low
		*Aphis gossypii*	19	Low
		*Acyrthosiphon caraganae*	10	Low
		*Chaitophorus leucomelas*	6	Low
		*Dysaphis plantaginea*	1	Low
	Eucalyptus tree honey (Calabria)	*Metcalfa pruinosa*	4127	High
		*Cinara cedri*	358	Medium
		*Aphis gossypii*	172	Low
		*Aphis taraxacicola*	70	Low
		*Cinara tujafilina*	70	Low
		*Brachycaudus cardui*	56	Low
		*Aphis spiraecola*	34	Low
		*Aphis craccivora*	9	Low
		*Aphis glycines*	1	Low
		*Brachycaudus persicae*	1	Low
		*Aphis egomae*	1	Low
		*Cervaphis rappardi*	1	Low
	Eucalyptus tree honey (Sicily)	*Metcalfa pruinosa*	2849	High
		*Myzus persicae*	32	Low
		*Acyrthosiphon pisum*	18	Low
		*Issus muscaeformis*	7	Low
		*Aphis gossypii*	1	Low
	French polyfloral honey (Corsica)	*Metcalfa pruinosa*	8675	High
		*Aphis gossypii*	145	Low
		*Aphis taraxacicola*	57	Low
		*Thelaxes suberi*	23	Low
		*Aphis ruborum*	10	Low
		*Issus coleoptratus*	4	Low
		*Dysaphis lappae*	3	Low
		*Aphis spiraecola*	2	Low
		*Acyrthosiphon malvae*	2	Low
		*Myzus persicae*	2	Low
		*Aphis glycines*	2	Low
		*Aphis craccivora*	2	Low
		*Cavariella aegopodii*	2	Low
		*Aulacorthum solani*	1	Low
		*Chaitophorus leucomelas*	1	Low
		*Issus muscaeformis*	1	Low
		*Hoplocallis picta*	1	Low
	Eastern Europe polyfloral honey	*Metcalfa pruinosa*	22856	High
		*Aulacorthum solani*	90	Low
		*Pemphigus bursarius*	46	Low
		*Eucallipterus tiliae*	4	Low
		*Hyperomyzus carduellinus*	1	Low

^a^Abundance category was assigned according to the proportion of reads over all Hemiptera reads identified in the analysed honey (see Methods).

#### Honeydew honeys

Of the five honeydew honeys analysed in this study, three (the oak honeydew honey produced in Lombardia region and the honeydew honeys produced in Trentino-Alto Adige and Veneto regions) showed the presence of just one Hemiptera species, *M*. *pruinosa*. This insect of the Flatidae family is a damaging polyphagous species which feeds on a variety of trees, crops and ornamental plants. The silver fir forest honey (another typical honeydew honey) produced in Emilia Romagna showed the presence of almost one Hemiptera species, *Cinara pectinatae* (99.9% of reads). Conversely only one read from this honey was assigned to *M*. *pruinosa*. The other silver fir forest honey produced in Piedmont showed about half of reads from *M*. *pruinosa* and half from *C*. *pectinatae*. *C*. *pectinatae* is an aphid known to feed on firs (*Abies* spp.), especially *Abies alba* (silver firs), congruent with these honeys’ botanical origin. In the later silver fir honey, a few other reads were attributed to other hemipters, the most represented of which were from *Issus muscaeformis* (2.89%) and *Cinara terminalis* (0.6%; Table 2).

#### Blossom honeys

*M*. *pruinosa* was also the only one species identified in the chestnut honey and the predominant hemipter in all other analysed blossom (or nectar) honeys as well. Reads assigned to this species in all remaining nectar honeys ranged from 64.98% in apple honey to 99.39% in Eastern Europe polyfloral honey (Fig. 1 and Table 2). Results reported below describe other hemipters with medium abundance and low abundance reads identified in the blossom honeys.

Apple tree honey produced in Trentino region (North of Italy, subalpine environment) showed the most heterogenous entomological signature with other 13 hemipter species (Fig. 1 and Table 2), seven of which (*Rhopalosiphum insertum*, *Myzus persicae*, *Cavariella aegopodii*, *Aphis gossypii*, *Therioaphis trifolii*, *Acyrthosiphon pisum* and *Aphis craccivora*) account for 1–15% of all sequenced reads obtained from this library. Among these Aphididae species, *R*. *insertum* (also known as *Rhopalosiphum oxyacanthae* or apple grass aphid) was the most represented (14.6% of assigned reads) providing an entomological and indirect signature of the botanical origin of this monofloral honey, considering the apple tree related biology of this aphid, confirmed by the metabarcoding analysis of the chloroplast *trnL-UAA* fragment. *M*. *persicae* (7.62%) and *A*. *gossypii* (3.76%) are polyphagus aphids feeding on a large number of plants whereas reads from *C*. *aegopodii* (4.45%; hosted mainly by Apiaceae and Salicaceae plants, the latter well represented from the metabarcoding analysis of the plant DNA), *T*. *trifolii* (1.38%; hosted by *Medicago* and *Trifolium* species), *A*. *pisum* (about 1%; mainly hosted by Fabaceae species) and *A*. *craccivora* (about 1%; polyphagus but with preference for legumes) provided a more detailed signature of the honey bee feeding environment and availability of honeydew from different botanical sources. Other detected aphid species, even if with a lower number of reads (<1%), confirmed the general signature of this honey produced in Trentino. For example, *Myzocallis castanicola* (sweet chestnut aphid) is mainly found on *Castanea sativa* (from which the insect species takes its name) and oaks that are quite common in the area from which the honey was produced. The absence of *Castanea* reads (a highly pollinic plant) in the apple tree blossom honey may be attributed to the different flowering period of the apple tree (end of April) and chestnut tree (end of May-beginning of June) in the region in which this honey was produced. Among other identified Hemiptera species, *Aphis citisorum* is mainly hosted by woody Fabaceae, *Brachycaudus salicinae* is usually found on *Inula salicina* (an Asteraceae plant frequently found in Trentino; from a plant family well represented among the reads of the plant metabarcoding analysis) and *Cinara pectinatae* is the silver fir aphid.

Linden tree (*Tilia* L.) honey (produced in the province of Udine, North-Eastern part of Italy) reported the presence of three Hemiptera species, each accounting for more than 1% of the total number of reads, and other six aphid species (or unspecified Aphididae species) accounting for a lower number of reads (Fig. 1 and Table 2). About 8.66% of the reads were from *Aphis craccivora*, 6.80% were from *Issus muscaeformis* and 2.77% were from *Eucallipterus tiliae*, an aphid species hosted by lime trees that supports the origin of the analysed honey. Other minor species were *Thelaxes suberi* (living on oaks, mainly on *Quercus cerris* and *Q*. *ilex*; family Fagaceae, that accounted the largest number of plant reads in the metabarcoding analysis), *Cinara cedri* (hosted by cedar and cypress), *Illinoia liriodendra* (hosted by the tulip tree, a.k.a. *Liriodendron tulipifera*, a North American tree introduced in Europe), *Aphis craccae* (hosted by legumes of the *Vicia* genus) and *Brachycaudus populi* (mainly found on *Silene* spp.).

Acacia honey (produced in Tuscany) reported reads assigned to several other aphids (in addition to *M*. *pruinosa*; Fig. 1 and Table 2). In particular, *Hyalopterus pruni* [hosted on giant cane (*Arundo donax*), common reeds (*Phragmites* spp.) and *Prunus* species, the latter well represented among the reads of the plant metabarcoding analysis] was the second most frequent hemipter identified in this honey, followed by *Aphis gossypii*, *Acyrthosiphon caraganae* (hosted by the Siberian peashrub, *Caragana arborescens*, an invasive ornamental species native to Asian), *Chaitophorus leucomelas* (found mainly on *Populus nigra* and related poplar species and hybrids) and *Dysaphis plantaginea* (hosted by apple trees and *Plantago* spp.).

The eucalyptus tree honey produced in Calabria region showed reads assigned to 11 hemipter species, in addition to *M*. *pruinosa* (Fig. 1 and Table 2). The most represented were *Cinara cedri* (hosted by several species of the *Cedrus* genus) that accounted 7.3% of reads, followed by *Aphis gossypii* (3.5%), *Aphis taraxacicola* (the dandelion aphid, hosted by *Taraxacum officinalis*; 1.4%), *Cinara tujafilina* (1.4%; hosted by several genera of Cupressaceae) and *Brachycaudus cardui* (1.1%; the thistle aphid that infests trees of the genus *Prunus* in spring and autumn and plants of the Asteraceae family in summer).

The eucalyptus tree honey produced in Sicily reported reads assigned to four hemipters, in addition to those derived by *M*. *pruinosa*: *Myzus persicae*, *Acyrthosiphon pisum* (feeding on plants of the family Fabaceae), *Issus muscaeformis* and *Aphis gossypii* (Fig. 1 and Table 2).

The polyfloral honey from the Corsica island (France) reported reads assigned to a total of 17 hemipter species (including *M*. *pruinosa*; Fig. 1 and Table 2) of which only *Aphis gossypii* showed >1% of reads. Other represented aphids were *Aphis taraxacicola*, *Thelaxes suberi* (hosted by *Quercus* spp., consistent with the botanical signature of this honey), *Aphis ruborum* (mainly hosted by blackberry) and *Issus coleoptratus* (a planthopper of the family Issidae that feeds on a variety of trees, i.e. *Acer* spp., *Corylus* spp., *Quercus* spp., *Tilia* spp., *Ulmus* spp.).

Few hemipter species were identified in the polyfloral Eastern Europe honey. A small fraction of total hemipter reads (<1%) were attributed to four aphids: *Aulacorthum solani*, hosted on potato, *Digitalis* spp. and several glasshouse plants; *Pemphigus bursarius*, hosted on poplar, lettuce and other Asteraceae species; *Eucallipterus tiliae*; *and Hyperomyzus carduellinus*, associated with *Sonchus oleraceus* and several other species (Fig. 1 and Table 2).

### Mitotype analysis from next generation sequencing data

The presence of mitochondrial haplotypes, or mitotypes, was evaluated by analyzing sequences obtained from Hemiptera species that produced a large number of reads in this study (i.e. *M*. *pruinosa*) or for which at least 10 reads were reported for at least two of the analysed honeys (i.e. *Myzus persicae* and *Aphis gossypii*). For each of these three species, more than 50 COI gene entries were available in GenBank/EMBL (Supplementary Tables S3 and S4).

**Figure 2:**
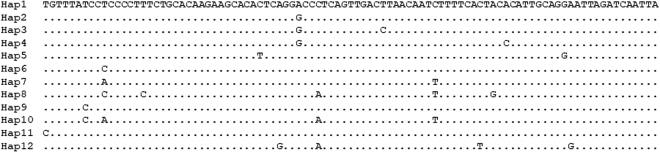
Sequence alignment of the M. pruinosa mitochondrial haplotypes. Nucleotide positions identical with those of the first sequence (Hap1) are marked with a dot.

A total of 136 *M*. *pruinosa* sequences were retrieved from these public repositories (Table 3 and Figure 2 and Supplementary Table S3). Most of these sequences were from a single study and were mostly collected in the Republic of Korea, but also from the United States, France, Italy, Spain and Slovenia^32^. In this study, Park *et al*.^32^ sequenced a larger COI gene region (470 bp; including the fragment targeted in this study) and reported the presence of 19 different mitotypes (H1-H19) based on the identified informative positions. However, considering only the shorter region used in this study, we could differentiate a total of 11 mitotypes. The correspondence between these mitotypes and those identified by Park *et al*.^32^ is reported in Supplementary Table S3.

**Table 3:** No. of reads assigned to the different *Metcalfa pruinosa* mitochondrial haplotypes (mitotypes) identified in the analysed honey samples compared to the haplotypes reported in GenBank entries. ^a^Sequence alignment of the listed haplotypes is reported in Figure 2. ^b^Number of reads corresponding to the identified mitotypes in the different honey samples indicated from 1 to 13: 1) Oak tree honeydew honey; 2) Honeydew honey (from Trentino Alto Adige); 3) Honeydew honey (from Veneto); 4) Silver fir honeydew honey (from Emilia-Romagna); 5) Silver fir honeydew honey (from Piedmont); 6) Chestnut tree honey; 7) Apple tree honey; 8) Linden/Lime tree (*Tilia*) honey; 9) Acacia honey; 10) Eucalyptus tree honey (from Calabria); 11) Eucalyptus tree honey (from Sicily); 12) French polyfloral honey (from Corsica): 13) Eastern Europe polyfloral honey. The bioinformatic method that was used to identify mitotypes was able to assign this information to most *M. pruinosa* reads reported in Table 2 for each analysed honeys. ^c^Number of *M. pruinosa* GenBank entries corresponding to the identified haplotypes. Correspondence with COI haplotypes reported by Park *et al*.^33^ is listed in Supplementary Table S3.

Haplotype ID^a^	No. of reads for each honey sample^b^													No. of entries^c^
	1	2	3	4	5	6	7	8	9	10	11	12	13	
Hap1	159	336	41	—	169	2073	43	175	306	396	153	379	2186	119
Hap2	—	—	—	—	—	—	—	—	—	—	—	—	—	2
Hap3	—	—	—	—	—	—	—	—	—	—	—	—	—	1
Hap4	—	—	—	—	—	—	—	—	—	—	—	—	—	1
Hap5	—	—	—	—	—	—	—	—	—	—	—	—	—	2
Hap6	5114	4318	3324	—	1075	11600	520	1474	1855	2802	2255	5659	15977	4
Hap7	—	—	—	—	—	—	—	—	—	—	—	—	—	2
Hap8	—	—	—	—	—	—	—	—	—	—	—	—	—	2
Hap9	—	—	—	—	—	—	—	—	—	—	—	—	—	1
Hap10	—	—	—	—	—	—	—	—	—	—	—	—	—	1
Hap11	—	—	—	—	—	—	—	—	—	—	—	—	—	1
Hap12	—	—	—	—	—	—	—	—	—	—	—	563	—	0

Reads matching the most frequent database mitotype (Hap1, reported in 119 entries and corresponding to H3, H8, H9, H15, H16, H18 or H19 of Park *et al*.^32^) were obtained in all honeys, except in the silver fir honeydew honey produced in Emilia Romagna. However, this mitochondrial haplotype (Hap1; Table 3 and Figure 2) was not the most frequent in all our samples (ranging from 6% to 15% of total *M*. *pruinosa* reads only). A mitotype already reported in GenBank/EMBL by four different entries (Hap6; corresponding to haplotypes H1 or H2 of Park *et al*.^32^) was the most frequent in all our honeys (from about 84% to 90% of reads). Another mitotype (not yet described by previous studies, according to publicly available sequences) was identified only in the Corsican honey that accounted 8.5% of total *M*. *pruinosa* reads.

Only one mitotype was identified for each of *Myzus persicae* and *Aphis gossypii*, and in all cases they corresponded to the more common mitotype based on the number of entries deposited in public DNA databases (Supplementary Table S4). It is worth mentioning that the number of sequences analysed for these species was smaller than those obtained for *M*. *pruinosa*.

## Discussion

Honey bees are regarded as large-scale monitoring tools that have been employed to detect industrial pollution and environmental contaminants found in hive components, including pollen, wax and honey^33,34^. Thus, honey can be considered an interesting source of environmental information, including environmental DNA, as its content reflects honey bee activities and foraging behavior and is influenced by complex biome and ecological networks. The combination of high throughput DNA metabarcoding approaches based on next generation sequencing and the unique environmental exploration capacity of honey bees makes it possible to disclose bio-ecological information that could not be otherwise obtained with any other system or tool. Honey bees can cover large areas and can easily sample in environments that are impractical or inaccessible using conventional sampling approaches. A few studies have already demonstrated the utility of pollen DNA analysis as a floral sampling detection system with potential applications for the identification of the botanical origin of honeys and the determination of honey bees foraging preferences^15–19^. Other uses of pollen DNA information could be for monitoring invasive plant species, for plant population genetic analyses, for biodiversity mapping and for floral characterization of inaccessible and extended areas^8,35,36^. A key element of plant metabarcoding is the use of universal primers able to amplify informative DNA regions. Several plastid and nuclear DNA regions have been tested for plant metabarcoding purposes^37^.

Following a similar strategy used for botanical identification, we designed PCR primers in conserved regions of Hemiptera species to amplify a short mtDNA COI gene fragment that was used to investigate DNA extracted from honey. Our study demonstrated that another important source of environmental DNA contained in honeys derives from honeydew produced by plant-sucking insects feeding on plants. These insects of the Hemiptera order excrete a carbohydrate-rich substance that can be used by honey bees to complement their nectar-based diet. Honeydew contains the DNA signature from the excreting insects that is not destroyed during the honey bee transformation processes, comb maturation or honey preservation. Therefore, Hemiptera DNA can be recovered and analysed to obtain information on the entomological origin of the honeydew fed by honey bees, providing an additional DNA signature that (indirectly) can contribute to understand the origin of the honey and authenticate this product. This aspect is worth of further investigation to evaluate if this signature could change in different production conditions and to understand if any factors influence the feeding behavior of the honey bees on honeydew.

Studies in other biological systems, from insects to mammals, have used DNA metabarcoding approaches to identify the species composition of the diet of different animals (e.g^38–43^.), with a direct identification on the DNA recovered from gut content or feces. In our case, we were able to go back on the feeding chain detecting the species (i.e. sucking insects) that transformed the plant sucked sap that in turn was fed and transformed by the honey bees. To compare the Hemiptera signature with the botanical composition and origin of the investigated honeys we also used the honey DNA for a plant metabarcoding analysis^20^. The botanical signature confirmed the prevalent botanical origin in many blossom unifloral honeys and provided other information that, in many cases, linked the plant-sucking insect signature with their plant hosts.

Even if the next generation sequencing approach that we used cannot be regarded as a quantitative approach, the number of assigned reads to a Hemiptera species might approximately indicate the relative abundance of the quantity of DNA derived by that species^23–26^. It is however important to critically consider this information for several reasons: i) amplification biases could be derived by priming preference of some taxonomic groups (for example, *M*. *pruinosa* might be preferentially amplified, as determined by qPCR analysis of insect DNA) and poor primer efficiency towards other species; ii) stochasticity during PCR amplification and the exponential nature of the PCR process, also taking into account the potential competition determined by the expected higher content of *Apis mellifera* DNA^9,12,13^; iii) the assignment to a species relies on the COI sequences being present in the database and incompleteness of insect COI database could limit the possibility to identify all possible species^44,45^; iv) it is not known the relationship between the amount of produced honeydew and the level of DNA of the producing hemipter in the excreted material; v) it is not known if there could be a different DNA degradation level derived by the honey bee transformation and subsequent maturation process depending on the source of honeydew and produced honey. Therefore, following Keller *et al*.^26^, abundance could be better interpreted as categorical (e.g. high abundance, medium abundance and low abundance) using the relative proportion (frequency) of reads over all sequenced reads without any linear association.

Considering these questions, it could be possible to indirectly define similar levels of feeding frequency or prevalence of the honey bees on the derived honeydew, based on the entomological signature in the honey. For example, the identification of almost exclusively or a high abundance of *Cinara pectinatae* sequences clearly support the origin of forest honeydew honey. Therefore, the identification of this aphid could be useful to overcome the limits of physical-chemical methods to distinguish honeydew honey from other honeys^46^. Another interesting entomological signature was obtained from the apple tree honey, produced in a particular ecological landscape (sub-alpine environment) that has greatly affected heterogeneity of aphid species, including apple tree specialized aphids (such as *Rhopalosiphum insertum*).

All analysed honeys (except a forest honeydew honey) reported the planthopper *M*. *pruinosa* as a high abundant species, that could be only in part derived by a higher amplification efficiency of its DNA using the designed primers. *M*. *pruinosa* is an invasive insect accidentally introduced in Italy in 1979 from the North America^46,47^ and is now present in several European countries and regions, mainly Austria, Bosnia, Bulgaria, Corsica, Croatia, Czech Republic, France, Greece, Germany Herzegovina, Hungary, Romania, Russia, Serbia, Slovenia, Spain, Switzerland and Turkey^48–50^ as well as Korea^51^. The geographical distribution of this pest might be useful for determining the geographical origin of honey. Its polyphagous sucking activity produces a high quantity of secreted honeydew, that in turn, becomes the substrate of mould that negatively impacts crop, tree and fruit production. Honeydew from *M*. *pruinosa* is considered an important carbohydrate source for honey bee colonies^52^ and the results we obtained suggested that honey bees usually find and feed on this resource, even when the availability of prevalent floral nectar offers an abundant alternative. The prevalence or, on the opposite side, the low abundance of *M*. *pruinosa* reads in honeys might open new interpretations and provide subsequent avenues for additional investigations. For example, it could be possible to relate this information to the health of colonies feeding on this carbohydrate rich resources and, at the same time, to monitor population dynamics of planthopper infestations over time and regions. It could also be informative to explore the relationship between *M*. *pruinosa* DNA abundance in honey and colony losses, as this problem seems tied to many different causes^53^ (e.g. pesticide use might indirectly affect honeydew production or lead to contaminated honeydew^54,55^).

This study creates new opportunities for monitoring the ecological interactions between plant sucking insects and their hosts and could help to develop new ecosystems models able to estimate the level of infestation of agricultural and forest landscapes. Retrospective evaluations could be obtained by analysing honey samples from colonies positioned in different areas and obtained from different times of the year. In this context, one limit could be the obscuring of other low abundance Hemiptera species by the large abundance of *M*. *pruinosa* DNA, precluding a more detailed overview of species without high sequencing depth (e.g. >20,000–50,000 reads for each library). This aspect is quite evident in the chestnut honey from which we obtained only *M*. *pruinosa* reads without any other reads that could be derived by other more specialized and informative Hemiptera species. For example, *M*. *castanicola*, the sweet chestnut aphid, expected also in the chestnut honey, was identified in the apple tree blossom honey from which a limited proportion of *M*. *pruinosa* reads was obtained. This technical aspect should be adjusted according to the needs and to the expected predominance of one hemipter species over others. Other universal Hemiptera primer pairs could be also designed and tested.

Analysis of DNA of entomological origin could be useful for monitoring invasive insect species (of the Hemiptera order), to infer the biology and the host range of aphids, to evaluate the entomological biodiversity and to indirectly infer the botanical composition of honey bee feeding areas, complementing direct analyses based on pollen metabarcoding. For several of the identified species (i.e. *Issus muscaeformis*) information on the biology, host preference, and geographical distribution is not clear.

The eDNA approach that we applied by targeting a mtDNA gene fragment provided useful information for population genetic studies of the most represented Hemiptera species. In general, these data could be useful to determine geographical provenance of subspecies or evaluate the distribution of ecotypes in relation to foraging behavior or for many other aspects related to the Hemiptera species under investigation. Again, this approach takes advantages from the broad sampling ability of the honey bees that could visit many sources of honeydews derived by different hemipter populations. Classical population genetic methods would collect individual insects and barcode them directly. Sampling and analytical costs could be very high, depending on the extension of the monitored areas and number of collected samples. Therefore, the indirect information that can be obtained from eDNA data might be cost-effective and could easily cover more extended regions.

We used sequence information of a few hemipter species to identify different mitochondrial haplotypes that could be detected among the sequenced reads. This study was informative in one of the three considered species (i.e. *M*. *pruinosa*). The abundance of reads that were assigned to the different *M*. *pruinosa* mitotypes could be interpreted as approximate indicator of the frequency of these variants in the covered areas, defined by the analysed honey. Park *et al*.^32^ reported that H1 was the predominant haplotype in most geographical regions and was the only one identified in Italy. We confirmed the prevalence of this haplotype in Italy (indicated in our study as Hap6; even if we could not distinguish it from H2, based on the shorter fragment we analysed; Table 3 and Figure 2) and identified another quite frequent haplotype (Hap1; that grouped several haplotypes described by Park *et al*.^32^; Supplementary Table S3). These results can open new opportunities to define the Italian biogeography structure of this invasive flatid planthopper species. A new *M*. *pruinosa* mitotype was identified in Corsica. Other studies are needed to evaluate its distribution in this island and in other countries.

## Conclusions

The unique entomological signature that derives from honeydew fed by honey bees opens new and interesting applications of environmental DNA contained in honey samples. Honey is one of the most frauded food products and the presence of DNA of some Hemiptera species creates new opportunities for honey authentication based on knowledge on the geographical distribution, biology, and host preference of these plant-sucking insects. Agricultural and forest landscapes are also influenced by these pests that should be monitored and then controlled. The combination of the unique environmental exploration capacity of honey bees, the possibility to recover environmental DNA from honey, and the unprecedent throughput of next generation sequencing technologies can produce a large amount of information useful for these purposes. Population genetic information derived by massive targeted resequencing data of Hemiptera species obtained in specifically designed and controlled experimental designs may also contribute to better understand population dynamics of damaging insect species. Other studies are needed to link these data and develop new models useful for applications in ecological and environmental entomology, pest management and honey bee feeding behavior monitoring.

## Electronic supplementary material

Supplementary Tables and Figures

Dataset 1
