# Supplementary material for: Entomological signatures in honey: an environmental DNA metabarcoding approach can disclose information on plant-sucking insects in agricultural and forest landscapes
Source: Sci Rep. 2018 Jul 3;8:9996. doi: 10.1038/s41598-018-27933-w (PMC6030050; doi:10.1038/s41598-018-27933-w)
Supplement: Supplementary file 1 — Supplementary Tables and Figures [file 41598_2018_27933_MOESM1_ESM.docx]

**Supplementary information**

**Entomological signatures in honey: an environmental DNA metabarcoding approach can disclose information on plant-sucking insects in agricultural and forest landscapes**

Valerio Joe Utzeri, Giuseppina Schiavo, Anisa Ribani, Silvia Tinarelli, Francesca Bertolini, Samuele Bovo, Luca Fontanesi

**Supplementary Table S1.**

Efficiency of qPCR amplification of DNA of several insect species (hemipters and honey bees) and honey samples based on the Ct value.

|  |  |  |  |
| --- | --- | --- | --- |
| **DNA sample/species** | **DNA concentration** | **Ct (mean ± s.d.)** | |
| *Metcalfa pruinosa* (specimen 1)^1^ | 6.00 ng/uL | 10.90 ± 0.52 | |
|  | 0.60 ng/uL | 13.97 ± 0.39 | |
|  | 0.06 ng/uL | 17.13 ± 0.41 | |
| *Metcalfa pruinosa* (specimen 2)^1^ | 6.00 ng/uL | 10.04 ± 0.39 | |
|  | 0.60 ng/uL | 12.95 ± 0.41 | |
|  | 0.06 ng/uL | 16.51 ± 0.47 | |
| *Issus muscaeformis* | 6.00 ng/uL | 21.81 ± 0.89 | |
|  | 0.60 ng/uL | 23.06 ± 0.79 | |
|  | 0.06 ng/uL | 26.09 ± 0.93 | |
| *Halyomorpha halys* | 6.00 ng/uL | 31.22 ± 0.93 | |
|  | 0.60 ng/uL | 34.65 ± 0.79 | |
|  | 0.06 ng/uL | 37.25 ± 0.98 | |
| *Aphis craccivora* | 6.00 ng/uL | 19.60 ± 0.48 | |
|  | 0.60 ng/uL | 22.54 ± 0.53 | |
|  | 0.06 ng/uL | 25.48 ± 0.49 | |
| *Cinara cedri* | 6.00 ng/uL | 17.74 ± 0.62 | |
|  | 0.60 ng/uL | 19.36 ± 0.49 | |
|  | 0.06 ng/uL | 23.44 ± 0.69 | |
| *Cinara cupressi* | 6.00 ng/uL | 20.25 ± 0.45 | |
|  | 0.60 ng/uL | 22.91 ± 0.77 | |
|  | 0.06 ng/uL | 26.65 ± 0.78 | |
| *Cinara pectinatae* | 6.00 ng/uL | 20.25 ± 0.58 | |
|  | 0.60 ng/uL | 22.91 ± 0.46 | |
|  | 0.06 ng/uL | 26.65 ± 0.89 | |
| *Myzus persicae* | 6.00 ng/uL | 18.56 ± 0.34 | |
|  | 0.60 ng/uL | 21.45 ± 0.47 | |
|  | 0.06 ng/uL | 24.65 ± 0.38 | |
| *Apis mellifera* (specimen 1)^1^ | 6.00 ng/uL | 27.23 ± 0.76 | |
|  | 0.60 ng/uL | 29.89 ± 0.59 | |
|  | 0.06 ng/uL | 32.30 ± 1.02 | |
| *Apis mellifera* (specimen 2)^1^ | 6.00 ng/uL | 26.98 ± 0.70 | |
|  | 0.60 ng/uL | 29.54 ± 0.60 | |
|  | 0.06 ng/uL | 32.15 ± 0.89 | |
| Oak tree honeydew honey | 6.00 ng/uL | 22.46 ± 0.78 | |
|  | 0.60 ng/uL | 24.90 ± 0.84 | |
|  | 0.06 ng/uL | 27.98 ± 0.79 | |
| Honeydew honey (Veneto) | 6.00 ng/uL | 33.63 ± 1.39 | |
|  | 0.60 ng/uL | 36.23 ± 1.27 | |
|  | 0.06 ng/uL | 38.90 ± 1.10 | |
| Eucalyptus tree blossom honey (Sicily) | 6.00 ng/uL | 31.81 ± 1.09 | |
|  | 0.60 ng/uL | 34.40 ± 1.17 | |
|  | 0.06 ng/uL | 37.45 ± 1.45 | |

^1^ Two different DNA specimens (vouchers) were tested for *M. pruinosa* and *A. mellifera*.

**Supplementary Table S2.**

Botanical groups identified in the analysed honeys using the metabarcoding method described in Utzeri *et al.*^20^.

| **Honey** | **Cluster ID number^1^** | **Taxa name** | **Taxa level** | **Number of reads^2^** |
| --- | --- | --- | --- | --- |
| Oak tree honeydew honey | 84 | *Castanea* | Genus | 450 |
|  | 8 | *Quercus* | Genus | 120 |
|  | 11 | Fagaceae | Family | 56 |
|  | 208 | Asteraceae | Family | 16 |
|  | 192 | *Trifolium* | Genus | 7 |
|  | 73 | Rosoideae | Subfamily | 6 |
| Honeydew honey (Trentino Alto-Adige) | 84 | *Castanea* | Genus | 489 |
|  | 192 | *Trifolium* | Genus | 23 |
|  | 73 | Rosoideae | Subfamily | 15 |
|  | 14 | Asteraceae | Family | 8 |
|  | 19 | *Pinus* | Genus | 5 |
|  | 42 | Poaceae | Family | 2 |
|  | 80 | *Prunus* | Genus | 2 |
| Honeydew honey (Veneto) | 192 | *Trifolium* | Genus | 457 |
|  | 84 | *Castanea* | Genus | 120 |
|  | 73 | Rosoideae | Subfamily | 87 |
|  | 147 | *Oryza* | Genus | 67 |
|  | 14 | Asteraceae | Family | 17 |
|  | 128 | Poeae | Tribe | 10 |
|  | 30 | Vitaceae | Family | 5 |
| Silver fir honeydew honey (Emilia Romagna) | 84 | *Castanea* | Genus | 1361 |
|  | 14 | Asteraceae | Family | 374 |
|  | 73 | Rosoideae | Subfamily | 353 |
|  | 134 | *Clematis* | Genus | 95 |
|  | 99 | *Abies* | Genus | 66 |
|  | 3 | Asteraceae | Family | 58 |
|  | 124 | Robinia | Genus | 35 |
|  | 97 | Papilionoideae | Subfamily | 35 |
|  | 26 | Asteroideae | Subfamily | 27 |
|  | 157 | *Plantago* | Genus | 27 |
|  | 180 | *Onopordium acanthium* | Species | 25 |
|  | 32 | Asteraceae | Family | 25 |
|  | 141 | *Dipsacus* | Genus | 23 |
|  | 80 | *Prunus* | Genus | 22 |
|  | 78 | Asteraceae | Family | 19 |
|  | 231 | *Hypericum* | Genus | 17 |
|  | 203 | *Rubus idaeus* | Species | 13 |
|  | 79 | Asteraceae | Family | 13 |
|  | 221 | *Ononis* | Genus | 12 |
|  | 252 | *Helianthemum apenninum* | Species | 12 |
|  | 74 | Lamiales | Order | 10 |
|  | 149 | Poinae | Subtribe | 10 |
|  | 251 | *Sorbus torminalis* | Species | 9 |
|  | 25 | Asteraceae | Family | 6 |
|  | 191 | Loteae | Tribe | 5 |
|  | 112 | Caesalpinioideae | Subfamily | 5 |
|  | 7 | *Fagus* | Genus | 4 |
|  | 1 | Cupressaceae | Family | 4 |
|  | 19 | *Pinus* | Genus | 4 |
|  | 194 | *Dactylis glomerata* | Species | 4 |
|  | 102 | *Populus* | Genus | 4 |
|  | 216 | Astereae | Tribe | 3 |
|  | 123 | *Hypochaeris tenuifolia* | Species | 3 |
|  | 113 | Caprifoliaceae | Family | 3 |
|  | 184 | Cupressaceae | Family | 3 |
|  | 68 | Sapindaceae | Family | 3 |
|  | 30 | Vitaceae | Family | 2 |
|  | 209 | *Chascolytrum brizoides* | Species | 2 |
|  | 208 | Asteraceae | Family | 2 |
|  | 207 | *Swartzia* | Genus | 2 |
|  | 233 | Malvoideae | Subfamily | 2 |
|  | 103 | *Tilia* | Genus | 2 |
|  | 61 | *Crepidiastrum ameristophyllum* | Species | 1 |
| Silver fir honeydew honey (Piedmont) | 84 | *Castanea* | genus | 608 |
|  | 252 | *Helianthemum apenninum* | Species | 67 |
|  | 80 | *Prunus* | Genus | 50 |
|  | 11 | Fagaceae | Family | 45 |
|  | 14 | Asteraceae | Family | 12 |
|  | 192 | *Trifolium* | Genus | 11 |
|  | 21 | *Quercus* | Genus | 10 |
|  | 208 | Asteraceae | Family | 2 |
| Chestnut tree blossom honey | 84 | *Castanea* | Genus | 1982 |
|  | 73 | Rosoideae | Subfamily | 526 |
|  | 147 | *Oryza* | Genus | 123 |
|  | 124 | *Robinia* | Genus | 122 |
|  | 35 | *Ficus* | Genus | 35 |
|  | 224 | *Oryza rufipogon* | Species | 25 |
|  | 37 | *Rosa* | Genus | 17 |
|  | 14 | Asteraceae | Family | 17 |
|  | 98 | Swartzieae | Tribe | 11 |
|  | 108 | Spiraeeae | Tribe | 11 |
|  | 71 | *Lagerostroemia* | Genus | 9 |
|  | 25 | Asteraceae | Family | 7 |
|  | 128 | Poeae | Tribe | 7 |
|  | 19 | *Pinus* | Genus | 7 |
|  | 112 | Caesalpinioideae | Subfamily | 6 |
|  | 16 | Asteraceae | Family | 6 |
|  | 3 | Asteraceae | Family | 6 |
|  | 239 | *Parietaria judaica* | Species | 6 |
|  | 12 | *Castanea crenata* | Species | 5 |
|  | 129 | Poeae | Tribe | 5 |
|  | 107 | Rhamneae | Tribe | 5 |
|  | 2 | Cannabaceae | Family | 5 |
|  | 216 | Astereae | Tribe | 5 |
|  | 143 | *Potentilla* | Genus | 4 |
|  | 53 | *Sambucus* | Genus | 4 |
|  | 43 | Ulmaceae | Family | 4 |
|  | 39 | *Magnolia* | Genus | 4 |
|  | 194 | *Dactylis glomerata* | Species | 3 |
|  | 30 | Vitaceae | Family | 3 |
|  | 42 | Poaceae | Family | 3 |
|  | 232 | *Lotus maritimus* | Species | 3 |
|  | 46 | *Morus* | Genus | 3 |
|  | 48 | Astereae | Tribe | 3 |
|  | 119 | Asteraceae | Family | 2 |
|  | 150 | *Fragaria viridis* | Species | 2 |
|  | 6 | Pooideae | Subfamily | 2 |
|  | 183 | *Glycine max* | Species | 2 |
|  | 27 | Campanulaceae | Family | 2 |
|  | 212 | *Galium* | Genus | 2 |
| Apple tree blossom honey | 49 | Rosaceae | Family | 1880 |
|  | 80 | *Prunus* | Genus | 1435 |
|  | 45 | Salicaceae | Family | 730 |
|  | 16 | Asteraceae | Family | 375 |
|  | 106 | *Pyrus* | Genus | 344 |
|  | 53 | *Sambucus* | Genus | 96 |
|  | 73 | Rosoideae | Subfamily | 96 |
|  | 67 | *Acer* | Genus | 87 |
|  | 124 | *Robinia* | Genus | 82 |
|  | 25 | Asteraceae | Family | 62 |
|  | 160 | Juglandaceae | Family | 36 |
|  | 68 | Sapindaceae | Family | 26 |
|  | 193 | *Liquidambar* | Genus | 23 |
|  | 48 | Astereae | Tribe | 18 |
|  | 72 | Betulaceae | Family | 17 |
|  | 104 | Maloideae | Subfamily | 9 |
|  | 46 | *Morus* | Genus | 9 |
|  | 203 | Rubus idaeus | Species | 9 |
|  | 13 | Betulaceae | Family | 9 |
|  | 115 | Asterales | Order | 6 |
|  | 79 | Asteraceae | Family | 5 |
|  | 51 | Asterales | Order | 4 |
|  | 253 | *Chiliadenus rupestris* | Species | 4 |
|  | 91 | Forsythieae | Tribe | 3 |
|  | 43 | Ulmaceae | Family | 3 |
|  | 107 | Rhamneae | Tribe | 3 |
|  | 1 | Cupressaceae | Family | 3 |
|  | 167 | Mutisioideae | Subfamily | 2 |
|  | 137 | Asteridae | Subclass | 2 |
|  | 5 | Lauraceae | Family | 2 |
|  | 41 | Magnoliaceae | Family | 2 |
|  | 90 | *Buxus* | Genus | 2 |
|  | 44 | *Ginkgo biloba* | Species | 2 |
|  | 83 | *Wisteria frutescens* | Species | 2 |
|  | 128 | Poeae | Tribe | 2 |
|  | 163 | *Poa* | Genus | 2 |
| Linden tree blossom honey | 11 | Fagaceae | Family | 16282 |
|  | 124 | *Robinia* | Genus | 132 |
|  | 80 | *Prunus* | Genus | 88 |
|  | 103 | *Tilia* | Genus | 37 |
|  | 194 | *Dactylis glomerata* | Species | 24 |
|  | 107 | Rhamneae | Tribe | 24 |
|  | 128 | Poeae | Tribe | 24 |
|  | 121 | Actinidiaceae | Family | 22 |
|  | 203 | *Rubus idaeus* | Species | 20 |
|  | 37 | *Rosa* | Genus | 20 |
|  | 19 | *Pinus* | Genus | 20 |
|  | 114 | *Buddleja* | Genus | 19 |
|  | 49 | Rosaceae | Family | 17 |
|  | 30 | Vitaceae | Family | 17 |
|  | 60 | *Clematis terniflora* | Species | 12 |
|  | 16 | Asteraceae | Family | 12 |
|  | 25 | Asteraceae | Family | 10 |
|  | 42 | Poaceae | Family | 9 |
|  | 14 | Asteraceae | Family | 9 |
|  | 67 | *Acer* | Genus | 9 |
|  | 160 | Juglandaceae | Family | 8 |
|  | 29 | Vitaceae | Family | 7 |
|  | 92 | *Ligustrum* | Genus | 6 |
|  | 102 | *Populus* | Genus | 6 |
|  | 66 | *Rorippa* | Genus | 5 |
|  | 220 | *Lonicera* | Genus | 5 |
|  | 129 | Poeae | Tribe | 5 |
|  | 191 | Loteae | Tribe | 5 |
|  | 149 | Poinae | SubTribe | 5 |
|  | 41 | Magnoliaceae | Family | 4 |
|  | 143 | *Potentilla* | Genus | 4 |
|  | 154 | *Begonia* | Genus | 4 |
|  | 65 | *Rorippa* | Genus | 4 |
|  | 81 | Lamiales | Order | 4 |
|  | 157 | *Plantago* | Genus | 4 |
|  | 182 | Lythraceae | Family | 3 |
|  | 252 | *Helianthemum apenninum* | Species | 3 |
|  | 119 | Asteraceae | Family | 3 |
|  | 151 | *Prunus cocomilia* | Species | 3 |
|  | 197 | *Polevansia rigida* | Species | 3 |
|  | 186 | Poaceae | Family | 3 |
|  | 51 | Asterales | Order | 3 |
|  | 185 | Solanoideae | Subfamily | 2 |
|  | 28 | *Potentilla* | Genus | 2 |
|  | 211 | Apioideae | Subfamily | 2 |
|  | 4 | *Rhododendron* | Genus | 2 |
|  | 93 | Verbeneae | Tribe | 2 |
|  | 6 | Pooideae | Subfamily | 2 |
|  | 183 | *Glycine max* | Species | 2 |
|  | 137 | Asteridae | Subclass | 2 |
|  | 222 | *Thesium* | Genus | 2 |
|  | 1 | Cupressaceae | Family | 2 |
|  | 217 | *Filipendula ulmaria* | Species | 2 |
|  | 200 | *Spiraea salicifolia* | Species | 2 |
|  | 3 | Asteraceae | Family | 2 |
|  | 79 | Asteraceae | Family | 2 |
| Acacia tree honey | 124 | *Robinia* | Genus | 2389 |
|  | 84 | *Castanea* | Genus | 719 |
|  | 37 | *Rosa* | Genus | 604 |
|  | 80 | *Prunus* | Genus | 338 |
|  | 112 | Caesalpinioideae | Subfamily | 310 |
|  | 49 | Rosaceae | Family | 301 |
|  | 16 | Asteraceae | Family | 187 |
|  | 8 | *Quercus* | Genus | 160 |
|  | 73 | Rosoideae | Subfamily | 134 |
|  | 45 | Salicaceae | Family | 130 |
|  | 67 | *Acer* | Genus | 97 |
|  | 25 | Asteraceae | Family | 65 |
|  | 53 | *Sambucus* | Genus | 56 |
|  | 131 | *Orobanche* | Genus | 37 |
|  | 19 | *Pinus* | Genus | 37 |
|  | 135 | Sanguisorbinae | Subtribe | 33 |
|  | 59 | Ebenaceae | Family | 23 |
|  | 121 | Actinidiaceae | Family | 15 |
|  | 30 | Vitaceae | Family | 12 |
|  | 188 | *Papaver* | Genus | 8 |
|  | 1 | Cupressaceae | Family | 8 |
|  | 203 | *Rubus idaeus* | Species | 8 |
|  | 227 | *Prunus webbii* | Species | 7 |
|  | 194 | *Dactylis glomerata* | Species | 7 |
|  | 36 | *Maclura* | Genus | 7 |
|  | 252 | *Helianthemum apenninum* | Species | 6 |
|  | 144 | *Oxalis* | Genus | 6 |
|  | 128 | Poeae | Tribe | 6 |
|  | 29 | Vitaceae | Family | 5 |
|  | 41 | Magnoliaceae | Family | 4 |
|  | 51 | Asterales | Order | 4 |
|  | 106 | *Pyrus* | Genus | 3 |
|  | 211 | Apioideae | Subfamily | 3 |
|  | 14 | Asteraceae | Family | 3 |
|  | 79 | Asteraceae | Family | 3 |
|  | 34 | Anacardiaceae | Family | 3 |
|  | 242 | *Cotinus coggygria* | Species | 3 |
|  | 245 | *Viburnum clemensae* | Species | 2 |
|  | 38 | *Allium* | Genus | 2 |
|  | 12 | *Castanea crenata* | Species | 2 |
|  | 83 | *Wisteria frutescens* | Species | 2 |
|  | 126 | Asteraceae | Family | 2 |
|  | 173 | *Acaena* | Genus | 2 |
|  | 55 | *Ranunculus* | Genus | 2 |
|  | 239 | *Parietaria judaica* | Species | 2 |
|  | 218 | *Viburnum* | Genus | 2 |
| Eucalyptus tree blossom honey (Calabria) | 50 | Myrtoideae | Subfamily | 795 |
|  | 228 | *Asparagus acutifolius* | Species | 234 |
|  | 84 | *Castanea* | Genus | 98 |
|  | 265 | Leptospermum | Genus | 89 |
|  | 86 | Myrtoideae | Subfamily | 87 |
|  | 73 | Rosoideae | Subfamily | 70 |
|  | 25 | Asteraceae | Family | 22 |
|  | 261 | Hibiscus | Genus | 11 |
|  | 56 | *Asparagus* | Genus | 8 |
|  | 6 | Pooideae | Subfamily | 2 |
| Eucalyptus tree blossom honey (Sicily) | 50 | Myrtoideae | Subfamily | 1842 |
|  | 228 | *Asparagus acutifolius* | Species | 682 |
|  | 84 | *Castanea* | Genus | 280 |
|  | 248 | *Syzygium pachyphyllum* | Species | 255 |
|  | 131 | *Orobanche* | Genus | 252 |
|  | 73 | Rosoideae | Subfamily | 165 |
|  | 180 | *Onopordium acanthium* | Species | 55 |
|  | 86 | Myrtoideae | Subfamily | 41 |
|  | 42 | Poaceae | Family | 39 |
|  | 19 | *Pinus* | Genus | 31 |
|  | 14 | Asteraceae | Family | 29 |
|  | 174 | *Pistacia mexicana* | Species | 26 |
|  | 25 | Asteraceae | Family | 22 |
|  | 144 | *Oxalis* | Genus | 13 |
|  | 26 | Asteroideae | Subfamily | 13 |
|  | 40 | Rutaceae | Family | 13 |
|  | 126 | Asteraceae | Family | 12 |
|  | 175 | Arundinoideae | Subfamily | 9 |
|  | 3 | Asteraceae | Family | 8 |
|  | 56 | *Asparagus* | Genus | 8 |
|  | 63 | *Capsicum* | Genus | 8 |
|  | 112 | Caesalpinioideae | Subfamily | 7 |
|  | 102 | *Populus* | Genus | 6 |
|  | 182 | Lythraceae | Family | 6 |
|  | 80 | *Prunus* | Genus | 6 |
|  | 37 | *Rosa* | Genus | 5 |
|  | 184 | Cupressaceae | Family | 5 |
|  | 170 | *Heliotropium* | Genus | 5 |
|  | 142 | *Cynodon* | Genus | 5 |
|  | 206 | *Pentanema cernuum* | Species | 5 |
|  | 78 | Asteraceae | Family | 4 |
|  | 87 | Myrtoideae | Subfamily | 3 |
|  | 230 | *Eryngium* | Genus | 3 |
|  | 121 | Actinidiaceae | Family | 3 |
|  | 111 | Caesalpinioideae | Subfamily | 3 |
|  | 160 | Juglandaceae | Family | 3 |
|  | 23 | Poaceae | Family | 3 |
|  | 57 | Convulvaceae | Family | 2 |
|  | 250 | *Kickxia* | Genus | 2 |
|  | 51 | Asterales | Order | 2 |
|  | 165 | *Holcus* | Genus | 2 |
|  | 47 | Lantaneae | Tribe | 2 |
|  | 67 | *Acer* | Genus | 2 |
|  | 6 | Pooideae | Subfamily | 2 |
|  | 202 | *Cedrus* | Genus | 2 |
|  | 2 | Cannabaceae | Family | 2 |
|  | 239 | *Parietaria judaica* | Species | 2 |
|  | 241 | *Lactuca inermis* | Species | 2 |
|  | 106 | *Pyrus* | Genus | 2 |
|  | 79 | Asteraceae | Family | 2 |
| Polyfloral honey (France, Corsica) | 84 | *Castanea* | genus | 574 |
|  | 50 | Myrtoideae | Subfamily | 234 |
|  | 8 | Quercus | genus | 100 |
|  | 11 | Fagaceae | Family | 49 |
|  | 19 | *Pinus* | Genus | 27 |
|  | 45 | Salicaceae | Family | 24 |
|  | 79 | Asteraceae | Family | 13 |
|  | 87 | Myrtoideae | Subfamily | 6 |
|  | 37 | *Rosa* | Genus | 3 |
| Polyfloral honey (Eastern Europe) | 80 | *Prunus* | Genus | 2742 |
|  | 49 | Rosaceae | Family | 739 |
|  | 45 | Salicaceae | Family | 649 |
|  | 67 | *Acer* | Genus | 509 |
|  | 183 | *Glycine max* | Species | 503 |
|  | 8 | *Quercus* | Genus | 265 |
|  | 124 | *Robinia* | Genus | 225 |
|  | 16 | Asteraceae | Family | 158 |
|  | 25 | Asteraceae | Family | 63 |
|  | 21 | *Quercus* | Genus | 61 |
|  | 53 | *Sambucus* | Genus | 47 |
|  | 84 | *Castanea* | Genus | 43 |
|  | 106 | *Pyrus* | Genus | 36 |
|  | 46 | *Morus* | Genus | 33 |
|  | 151 | *Prunus cocomilia* | Species | 33 |
|  | 243 | *Crataegus monogyna* | Species | 29 |
|  | 37 | *Rosa* | Genus | 22 |
|  | 190 | *Staphylea* | Genus | 20 |
|  | 149 | *Poinae* | Genus | 13 |
|  | 10 | *Picaea* | Genus | 11 |
|  | 13 | Betulaceae | Family | 8 |
|  | 177 | Salicaceae | Family | 7 |
|  | 42 | Poaceae | Family | 7 |
|  | 112 | Caesalpinioideae | Subfamily | 7 |
|  | 73 | Rosoideae | Subfamily | 6 |
|  | 159 | *Barbarea vulgaris* | Species | 5 |
|  | 14 | Asteraceae | Family | 5 |
|  | 76 | Cardamineae | Tribe | 4 |
|  | 197 | *Polevansia rigida* | Species | 4 |
|  | 129 | Poeae | Tribe | 4 |
|  | 48 | Astereae | Tribe | 4 |
|  | 3 | Asteraceae | Family | 4 |
|  | 1 | Cupressaceae | Family | 3 |
|  | 58 | Brassicaceae | Family | 2 |
|  | 77 | Brassicaceae | Family | 2 |
|  | 195 | Ranunculus | Genus | 2 |
|  | 185 | Solanoideae | Subfamily | 2 |
|  | 52 | Celastraceae | Family | 2 |
|  | 103 | Tilia | Genus | 2 |

^1^ ID of the botanical group as defined in the customized database.

^2^ Only botanical groups including at least two reads were reported.

**Supplementary Table S3.**

Correspondence between *Metcalfa pruinosa* mitotypes identified in our study and those reported in Park *et al.*^33^ and those deduced from GenBank mining of sequences of this planthopper species. Other details are reported in Table 3.

| **Haplotype ID** | **Haplotypes reported by Park *et al.*** | **GenBank entry^1^** | **Number of GenBank entries ^2^** |
| --- | --- | --- | --- |
| Hap1 | H3, H8, H9, H15, H16, H18, H19 | KT382718 | 119 |
| Hap2 | H11, H13 | KT382708 | 2 |
| Hap3 |  | KT382709 | 1 |
| Hap4 | H10 | KJ412927 | 1 |
| Hap5 | H4 | KJ412949 | 2 |
| Hap6 | H1, H2 | KT382545 | 4 |
| Hap7 |  | KR038483 | 2 |
| Hap8 |  | KR038966 | 2 |
| Hap9 |  | KJ412931 | 1 |
| Hap10 |  | KJ412925 | 1 |
| Hap11 |  | KJ412928 | 1 |
| Hap12 |  | - | 0 |

^1^ GenBank entries reported as example of the mitotypes.

^2^ Number of entries already deposited in GenBank by others and corresponding to the identified mitotypes.

**Supplementary Table S4.**

*Aphis gossypii* and *Myzus persicae* COI gene mitotypes identified in GenBank and number of reads identified in different honey samples corresponding to the reported mitotypes.

| ***Aphis gossypii* GenBank entry^1^** | **Mytotype ID** | **Number of GenBank entries ^2^** | **Number of reads in apple tree honey** | **Number of reads in acacia honey** | **Number of reads in Eucalyptus tree honey (Calabria)** | **Number of reads in French poly-floral honey (Corsica)** |
| --- | --- | --- | --- | --- | --- | --- |
| KP152417 | Hap_Aph1 | 5 |  |  |  |  |
| KY842560 | Hap_Aph2 | 158 | 36 | 19 | 170 | 140 |
| KP152479 | Hap_Aph3 | 1 |  |  |  |  |
| JQ690335 | Hap_Aph4 | 1 |  |  |  |  |
| JQ690333 | Hap_Aph5 | 1 |  |  |  |  |
| JQ067097 | Hap_Aph6 | 1 |  |  |  |  |
| JX966028 | Hap_Aph7 | 1 |  |  |  |  |
| KP152451 | Hap_Aph8 | 1 |  |  |  |  |
| KP152429 | Hap_Aph9 | 1 |  |  |  |  |
| KM268006 | Hap_Aph10 | 1 |  |  |  |  |
| JQ690329 | Hap_Aph11 | 2 |  |  |  |  |
| AB506729 | Hap_Aph12 | 1 |  |  |  |  |
| KT356160 | Hap_Aph13 | 2 |  |  |  |  |
| FJ965683 | Hap_Aph14 | 1 |  |  |  |  |
| KP152444 | Hap_Aph15 | 1 |  |  |  |  |
| KP152430 | Hap_Aph16 | 1 |  |  |  |  |
| KF446152 | Hap_Aph17 | 1 |  |  |  |  |
| AY227082 | Hap_Aph18 | 1 |  |  |  |  |
| EU701359.1 | Hap_Aph19 | 1 |  |  |  |  |
| KJ502183.1 | Hap_Aph20 | 1 |  |  |  |  |
| ***Myzus persicae* GenBank entry** | **Mytotype ID** | **Number of GenBank entries ^2^** | **Number of reads in apple tree honey** | **Number of reads in Eucalyptus tree honey (Calabria)** |  |  |
| KX054006 | Hap_Myz1 | 61 | 63 | 30 |  |  |
| KT356173 | Hap_Myz2 | 1 |  |  |  |  |
| JX051436 | Hap_Myz3 | 1 |  |  |  |  |
| DQ871252 | Hap_Myz4 | 1 |  |  |  |  |
| AB506739 | Hap_Myz5 | 1 |  |  |  |  |

^1^ GenBank entries reported as example of the mitotypes.

^2^ Number of entries deposited in GenBank corresponding to the identified mitotypes.

**Supplementary Figure S1**

Alignment of a few sequences corresponding to the amplified region from Hemiptera species of different suborders and families (sequences included in the alignment are from plant-sucking species producing honeydew that are representative of the family; primer regions are coloured). *Cinara cedri* sequence has been selected as reference in this alignment.

**Supplementary Figure S2**

Alignment of the *Cinara cedri* analysed COI sequence region (from KU754492) with the corresponding region of *Apis mellifera ligustica* (from KF833391).

***Cinara cedri* 1 TGGAACAGGAACAGGATGAACAATTTATCCCCCCTTATCAAATAATATTGCACATAATAATAT---TTCAGTAGATTTAACTATCTTCTCTCTCCATTTAGCAGGAATTTCATCAATTTTAGGAGCAATCAATTTTATTT 137**

***Apis mellifera* 313 AA..C.......T.........G.A.....A..A......GC--.....T-.T..C..TC.TCACC............T..A..T..T.....T...A..T..........C......A.....T..T.A.....A..AG 447**

**Supplementary Dataset S1 online.**

Alignments of the forward and reverse primers with 5000 Hemiptera entries (Excel file).
